# Pathological progress and remission strategies of osteoarthritic lesions caused by long-term joint immobilization

**DOI:** 10.1186/s13075-023-03223-3

**Published:** 2023-12-07

**Authors:** Donghao Gan, Xiaowan Jin, Xiangpeng Wang, Chu Tao, Qinnan Yan, Qingyun Jia, Shaochuan Huo, Di Chen, Qing Yao, Guozhi Xiao

**Affiliations:** 1 Department of Biochemistry, Guangdong Provincial Key Laboratory of Cell Microenvironment and Disease Research, Shenzhen Key Laboratory of Cell Microenvironment, School of Medicine, Southern University of Science and Technology, Shenzhen, China; 2 First College of Clinical Medicine, Shandong University of Traditional Chinese Medicine, Jinan, China; 3 Department of Orthopedics, Linyi People’s Hospital, Linyi, China; 4 Shenzhen Hospital (Futian) of Guangzhou University of Chinese Medicine, Shenzhen, China; 5 Research Center for Computer-Aided Drug Discovery, Shenzhen Institute of Advanced Technology, Chinese Academy of Sciences, Shenzhen, 518055 China

**Keywords:** Osteoarthritis, Joint immobilization, Remobilization, Synovial invasion, Osteophytes

## Abstract

**Objective:**

While joint immobilization is a useful repair method for intra-articular ligament injury and periarticular fracture, prolonged joint immobilization can cause multiple complications. A better understanding how joint immobilization and remobilization impact joint function and homeostasis will help clinicians develop novel strategies to reduce complications.

**Design:**

We first determined the effects of long-term immobilization on joint pain and osteophyte formation in patients after an extraarticular fracture or ligament injury. We then developed a mouse model of joint immobilization and harvested the knee joint samples at 2, 4, and 8 weeks. We further determined the effects of remobilization on recovery of the osteoarthritis (OA) lesions induced by immobilization in mice.

**Results:**

We found that the long-term (6 weeks) joint immobilization caused significant joint pain and osteophytes in patients. In mice, 2-week immobilization already induced moderate sensory innervation and increased pain sensitivity and infiltration in synovium without inducing marked osteophyte formation and cartilage loss. Long-term immobilization (4 and 8 weeks) induced more severe sensory innervation and inflammatory infiltration in synovium, massive osteophyte formation on both sides of the femoral condyle, and the edge of the tibial plateau and significant loss of the articular cartilage in mice. Remobilization, which ameliorates normal joint load and activity, restored to certain extent some of the OA lesions and joint function in mice.

**Conclusions:**

Joint immobilization caused multiple OA-like lesions in both mice and humans. Joint immobilization induced progressive sensory innervation, synovitis, osteophyte formation, and cartilage loss in mice, which can be partially ameliorated by remobilization.

**Supplementary Information:**

The online version contains supplementary material available at 10.1186/s13075-023-03223-3.

## Introduction

Joint immobilization is a common repairmethod for intra-articular ligament injury and periarticular fracture. Complications, such as joint stiffness and osteoarthritis (OA), are common after prolonged joint immobilization, which may lead to severe pain and disability [1]. Clinical and histological studies have shown that stress imbalance in articular cartilage is an important factor in the progression of OA [2, 3]. Moderate joint load and exercise are essential for maintaining articular cartilage structure and function [4]. Some studies showed that intermittent hydrostatic pressure can maintain cartilage function. In the absence of joint load, it manifests as cartilage disuse lesions with thinning of articular cartilage and decreased matrix staining, while excessive mechanical stress can also promote the progression of OA [5].

Intractable pain and accelerated joint degeneration are the main problems urgently to be solved in clinical practice [6, 7]. Some studies showed that synovitis is the direct cause of pain, but the correlation between synovitis and pain and joint degeneration after long-term joint immobilization is not very clear [8, 9]. In addition, osteophytes are also associated with functional disability [10]. Therefore, further efforts are still needed to discover the pathological mechanisms of OA induced by joint immobilization. In addition, the strategy of restoring joint structure and function depends greatly on the understanding of the pathological changes of the joint and the progression of motor inhibition pathology.

The objectives of this study were to determine the effects of knee joint immobilization and remobilization on joint homeostasis at the molecular, histological, and animal levels as well as in human patients to provide useful information for clinicians to develop novel strategies to reduce the complications of joint immobilization.

## Materials and methods

### Patients

Clinical observation analyses were performed on patients with an extraarticular fracture or ligament injury treated with joint immobilization, such as plaster external immobilization in the Linyi People’s Hospital. Visual analogue scale (VAS) score, which measures pain intensity, and X-ray imaging were analyzed before, after surgery, and during follow-up, to evaluate joint pain and observe osteophyte formation at the joint site. This clinical observation study was approved by the Ethics Committee of Linyi People’s Hospital (no. YX200278).

### Animals

Forty-two C57BL6/J mice (male, 8 weeks old) were purchased from the Model Animal Research Center of Nanjing University (Nanjing, China). The mice were housed in a specific pathogen-free (SPF) facility at the Southern University of Science and Technology of China. The experimental protocol was approved by the Institutional Animal Care and Use Committee of the Southern University of Science and Technology (SUSTech-JY202108032). The mice were randomly divided into experimental group and a control group (*n* = 6 per group, male). The experimental group mice were subjected to knee joint immobilization or remobilization after 2 weeks of immobilization. No animals were excluded from the study due to adverse effects.

### Construction of mouse model

Plaster knee joint immobilization in mice was established according to a previously described protocol in rabbits [11]. Briefly, two layers of plaster were soaked in warm water for 30 s, and the inner layer of plaster was covered with gauze and placed under the right leg of mice to wrap and fix the right leg.

### Micro-computed tomography

The mouse knee joints were fixed with 4% paraformaldehyde and scanned using a SkyScan 1276 high-resolution CT scanner (Bruker) with a voltage of 60-kV and 100-uA current. 3D reconstruction and data analysis were then performed [12].

### Animal behavioral tests

Testing for mechanical allodynia (von Frey sensitivity) was performed according to the method we described previously [13]. Mice were first placed on an elevated mesh platform and acclimated for 15 min in a quiet environment. The hind paw was then dialed from below using calibrated von Frey wire and calculated using an iterative method.

### Histological analyses

Tissue specimens were fixed in 4% paraformaldehyde at 4 °C for 24 h. Specimens were continuously decalcified with 10% EDTA (pH 7.4) for 2 weeks at 4 °C, and then the specimens were paraffin embedded, and 5-μm-thick sagittal sections were cut. Hematoxylin-eosin (H/E), safranin O/fast green (SO/FG) staining, and Masson trichrome staining were performed as previously described [14, 15]. Cartilage degeneration was assessed by SO/FG staining using the Osteoarthritis Research Society International (OARSI) scoring system [16]. H/E sections were used to assess synovial activation through Krenn’s synovitis score system and to assess osteophyte size and maturity [17, 18]. Semi-quantification of synovial fibrosis was evaluated by calculating the percentage of collagen I-positive areas with ImageJ software. Each section was evaluated by two blind independent raters, and the mean scores were used for statistical analysis.

### Collagen analysis by two-photon laser microscope

Two-photon fluorescence signals from each sample were collected using a two-photon laser microscope (Olympus FVMPE-RS), and the Fiji ImageJ software was used to analyze the mean signal intensity and the distribution of collagen fibers in the synovium [19].

### Immunofluorescence and confocal analysis

Specimens were prepared as previously described [20]. Sections were antigen retrieved overnight in sodium citrate buffer, permeabilized sections with 0.2% Triton X-100, blocked with 2% BSA for 1 h, and then incubated with primary antibodies overnight at 4 °C. After washing, sections were incubated with anti-rabbit Alexa Fluor 488 (Invitrogen) secondary antibodies (1:400 dilution) for 1 h at room temperature. The fluorescence signal of the region of interest was determined using a confocal microscope (ZEISS Confocal Microscopy System). Semiquantitative analysis of immunofluorescence (IF) staining was performed in a double-blind manner.

### Immunohistochemistry

Briefly, after antigen retrieval, hydrogen peroxide treatment, BSA blocking, and primary antibody incubation overnight, the slices were incubated with horseradish peroxidase (HRP) combined with the second antibody and then incubated again with DAB staining (Abcam) and hematoxylin restaining. Finally, the images were captured, and semiquantitative analysis was analyzed using Fiji-ImageJ software.

### Statistical analysis

All experiments were performed by two investigators blinded to the experimental grouping. All data were presented as mean ± standard deviation and analyzed or plotted using GraphPad Prism 8.0. Differences between the two groups were analyzed using Student’s *t*-test. For comparisons between more than two groups, one-way analysis of variance (ANOVA) was used. Significance was defined as *P* < 0.05.

## Results

### Joint immobilization causes joint pain and osteophyte formation in the knee joint in patients

**Fig. 1:**
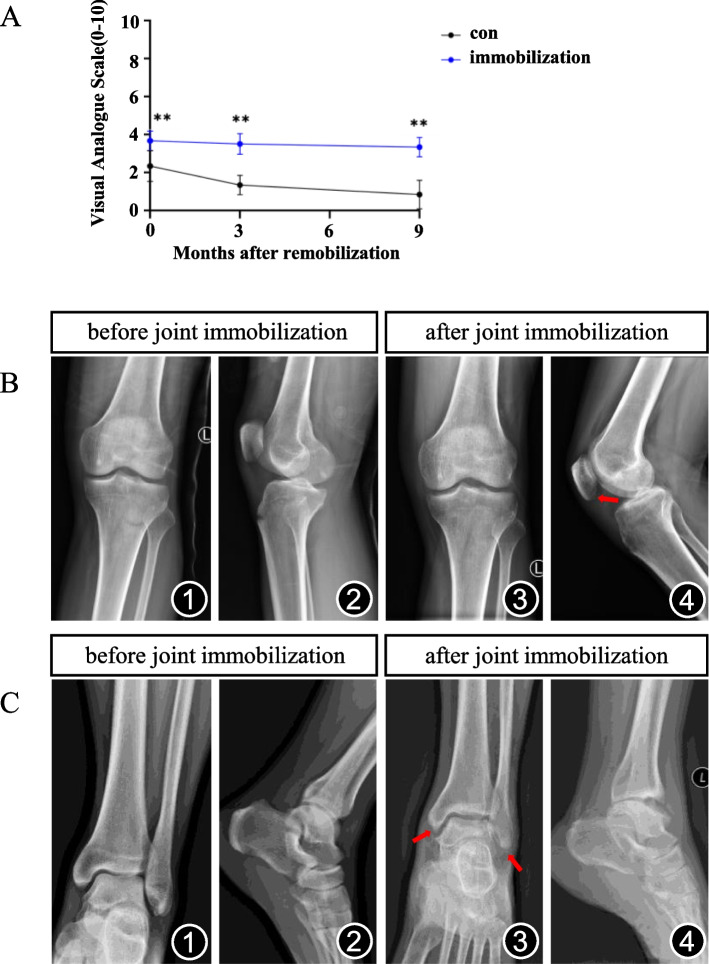
Joint immobilization causes joint pain and osteophyte formation in patients. **A** Visual analogue scale (VAS) scores. VAS scores were obtained in patients with extraarticular fractures with and without 6-week immobilization at the indicated time points after mobilization removal. *N* = 6 per group. Results were expressed as mean ± standard deviation (sd). **P < 0.01. **B** Typical case 1. The patient underwent conservative external fixation for avulsion fracture of the left tibial tubercle, which was removed 6 weeks later. The patient was unable to move due to knee pain, and X-ray examination showed slight osteophyte of patella. Anterior and lateral X-ray of knee joints at the beginning of immobilization (B1, B2), anterior and lateral X-ray of knee joints 6 weeks after joint immobilization (B3, B4). **C** Typical case 2. The patient underwent conservative treatment with external fixation for avulsion fracture of the left lateral ankle, which was excluded after 6 weeks. Due to ankle pain, the patient was afraid to move, and X-ray examination showed serious osteophyte formation 1 year later. Anterior and lateral X-ray of ankle joints before joint immobilization (C1, C2), anterior and lateral X-ray of ankle joints 1 year after joint immobilization (C3, C4). The red arrow shows osteophyte

The results from our follow-up studies showed that patients who were treated with long-term (6-week) joint immobilization for extraarticular fractures had experienced more joint stiffness and limitation of motion after the joint remobilization, and the visual analogue scale (VAS) scores, which measures pain intensity, were time dependently increased compared to controls after the joint immobilization removal (Fig. 1A). The images from a patient who underwent conservative external fixation for 6 weeks for avulsion fracture of the left tibial tubercle showed a slight osteophyte of patella appeared after 6-week joint immobilization (Fig. 1B). Another patient who underwent external fixation (6 weeks) for avulsion fracture of the left lateral ankle showed osteophyte formation on the edge of ankle joint (Fig. 1C).

### Joint immobilization promotes massive osteophyte formation and ossification of meniscus in the knee joint in mice

**Fig. 2:**
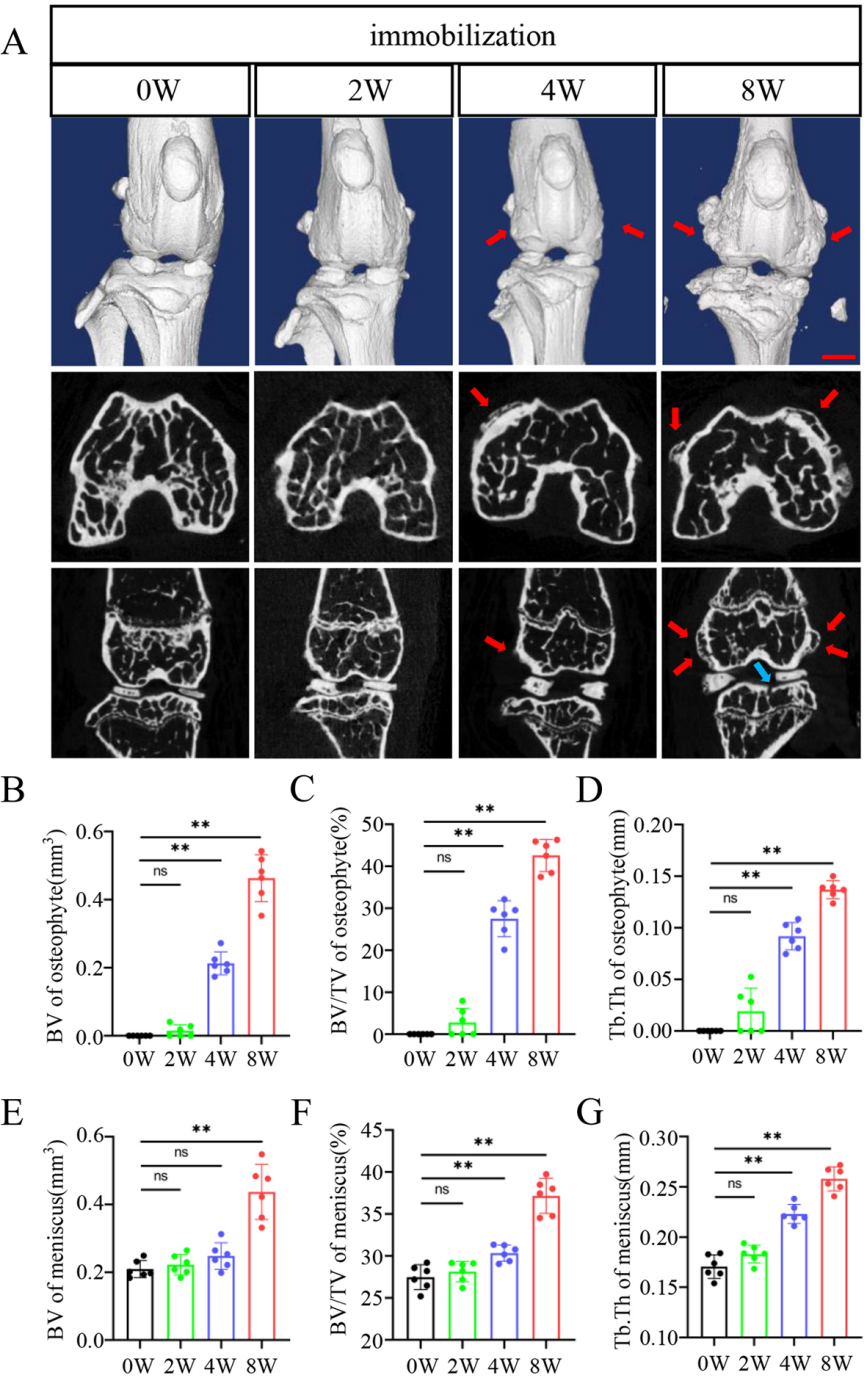
Joint immobilization stimulates osteophyte formation in mice. **A** Three-dimensional (3D) and two-dimensional (2D) reconstruction of μCT scans of knee joints at 0, 2, 4, and 8 weeks after immobilization. The first row is the 3D reconstruction front view of the knee joint; the second row is the 2D cross-sectional view of the femoral condyle, and the third row is the 2D front view of the knee joint. The red arrowheads show osteophytes of the femoral condyle, and blue arrowheads show microfracture in the tibial plateau. Scale bar, 1.0 mm. **B**–**D** The bone volume (BV), bone volume/total volume (BV/TV), and trabecular thickness (Tb.Th) of osteophytes on both sides of femoral condyle were analyzed by μCT. **E**–**G** The BV, BV/TV, and Tb.Th of calcified meniscus were analyzed by μCT. *N* = 6 biologically independent replicates per group. Results were expressed as mean ± standard deviation (sd). **P < 0.01

We used plaster to fix the knee joint of mice to establish the lower limb extension immobilization model (Supplementary Fig. 1A). μCT analyses revealed no obvious abnormalities in the knee joint of mice with 2 weeks of immobilization. After 4 weeks of immobilization, there was osteophytes formation on both sides of the femoral condyle (Fig. 2A–D) and obvious ossification of meniscus (Fig. 2A, E–G). After 8 weeks of immobilization, there were massive osteophytes formed on both sides of the femoral condyle, sclerosis of the bone cortex (Fig. 2A, B–D), obvious ossification of the meniscus, and micro fractures on the tibial plateau (Fig. 2A, E–G).

### Joint immobilization promotes progressive synovial infiltration, articular cartilage loss, vascular formation, and pain in mice

**Fig. 3:**
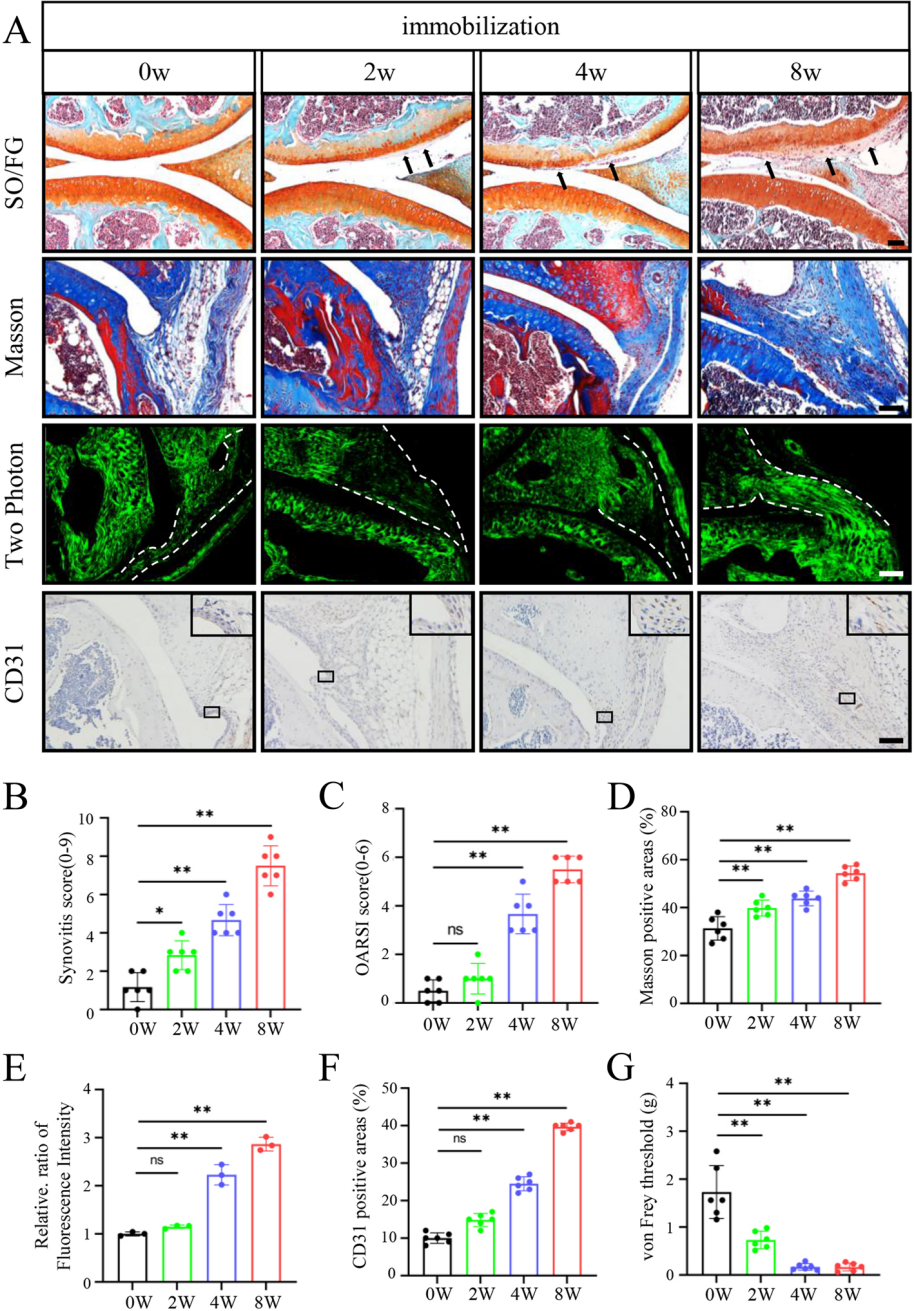
Joint immobilization induces synovial hyperplasia and fibrosis and promotes cartilage lesions in mice. **A** Representative images of safranin O/fast green (SO/FG) staining (first row) in articular cartilage, Masson staining (second row), two photon imaging for collagenous fiber (third row) and immunohistochemical (IHC) staining of CD31 (fourth row) in synovial tissues of knee joints at 0, 2, 4, and 8 weeks after immobilization. The black arrowheads represent the invading synovium; white dashed lines indicate the synovial tissue. Scale bar, 50 mm. **B** Synovitis score based on staining results in **A**. **C** OARSI score based on staining results in **A**. **D** Percentage of type 1 collagen-positive area based on Masson staining results in **A**. **E** Percentage of type 1 collagen-positive area based on two-photon results in **A**. **F** Percentage of CD31-positive area based on IHC staining results in **A**. **G** Results of von Frey test. *N* = 6 biologically independent replicates per group. Results were expressed as mean ± standard deviation (sd). *P < 0.05, **P < 0.01

Results from SO/FG staining of joint sections showed slight synovial infiltration after 2 weeks of immobilization compared to that in control group. After 4 weeks of immobilization, the synovial lining cells invaded the joint cavity along the cartilage surface, and the periarticular cartilage was eroded by the synovium. After 8 weeks of immobilization, hyperplasia of synovium was observed around the joint, and the synovial lining cells eroded articular cartilage (Fig. 3A–C). Masson staining and two-photon imaging showed synovial tissue of the immobilization models showed deeper collagen staining than that of the control, and the percentage of type 1 collagen-positive area was increased with the extension of immobilization time. The intensity of collagen fibers at the junction of synovial tissue and osteophyte was significantly increased by immobilization (Fig. 3A, D, E). Joint immobilization induced the vascular formation in synovium, as demonstrated by increased CD31 staining, and induced significant pain, as measured by von Frey test (Fig. 3A, F, G).

### Joint immobilization stimulates sensory innervation in synovium in mice

**Fig. 4:**
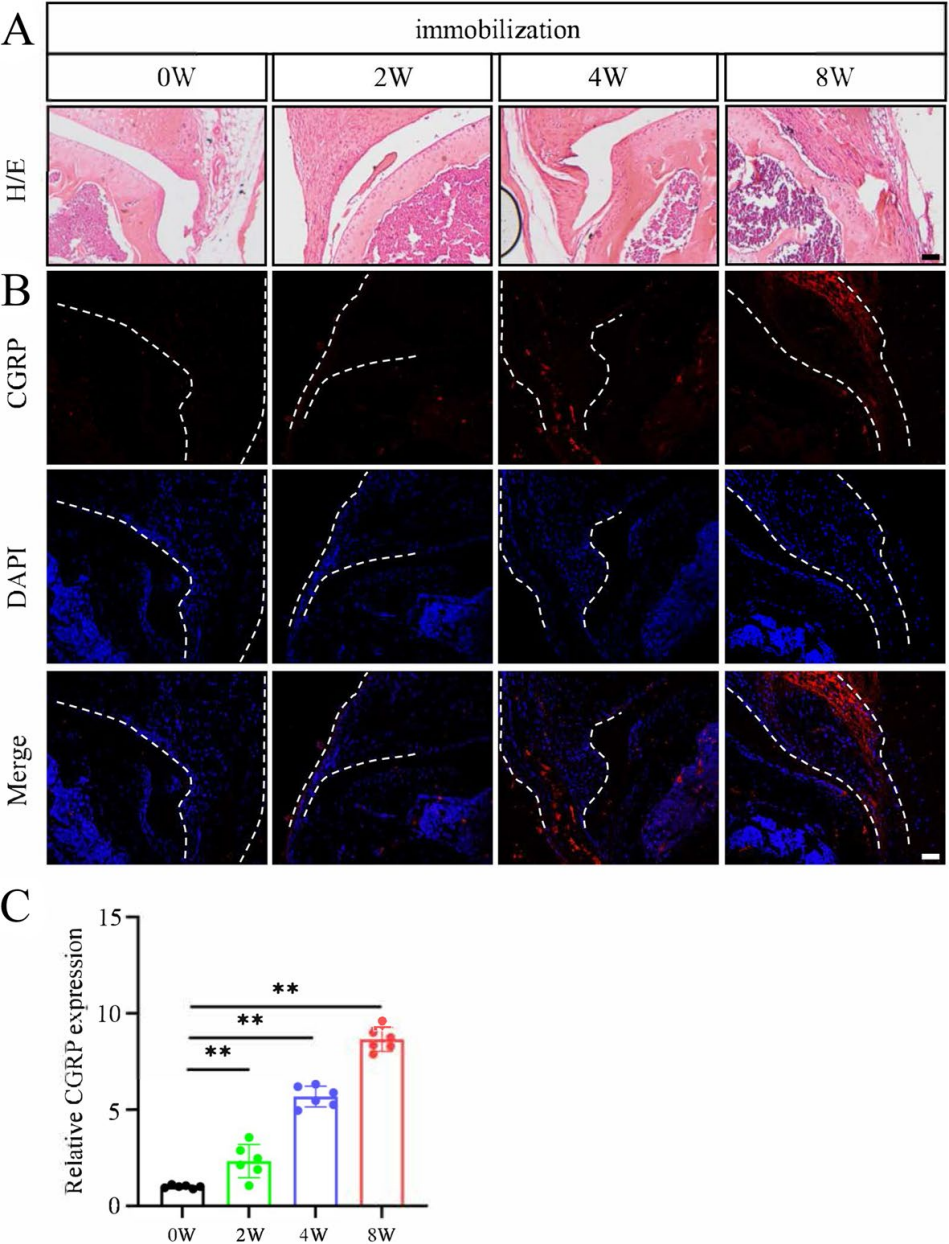
Joint immobilization induces sensory innervation in synovium in mice. **A** Representative images of H/E staining images in knee joints at 0, 2, 4, and 8 weeks after immobilization. Scale bar, 50 mm. **B** Representative images of immunofluorescence (IF) staining of CGRP staining (first row), DAPI staining (second row) and merged image (third row) in synovial tissue of knee joints at different groups. The white dashed lines indicate the synovial tissue. Scale bar, 50 mm. **C** Quantitative data of expression of CGRP based on staining results in **B**. *N* = 6 biologically independent replicates per group. Results were expressed as mean ± standard deviation (sd). **P < 0.01

To further investigate the correlation between pain sensitivity and sensory innervation in synovium after immobilization, we detected pain-related calcitonin gene-related peptide (CGRP) expression level in synovium tissues of four groups. IF staining showed that joint immobilization time dependently increased the expression level of CGRP protein in the synovium (Fig. 4).

### Joint remobilization ameliorates some OA lesions and synovial sensory innervation caused by immobilization in mice

**Fig. 5:**
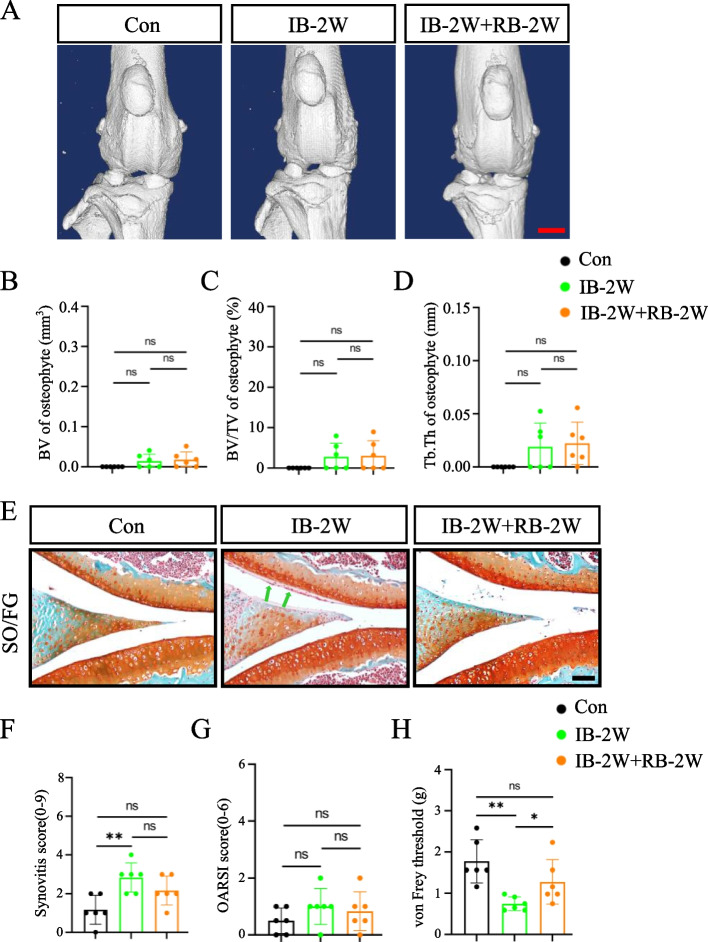
Joint remobilization ameliorates OA lesions caused by short-term immobilization in mice. **A** 3D reconstruction from μCT scans of knee joints from control, immobilization for 2 weeks, remobilization after 2 weeks of immobilization. Scale bar, 1.0 mm. **B**–**D** The BV, BV/TV, and Tb.Th of osteophytes on both sides of femoral condyle were analyzed by μCT. **E** Representative images of SO/FG staining images in knee joints of different groups. Green arrowheads show synovial hyperplasia. Scale bar: 50 mm. **F** and **G** Synovitis score (**F**), OARSI score (**G**) based on staining results in (**E**). **H** Results of von Frey test. *N* = 6 biologically independent replicates per group. Results were expressed as mean ± standard deviation (sd). *P < 0.05, **P < 0.01. IB, immobilization; RB, remobilization

**Fig. 6:**
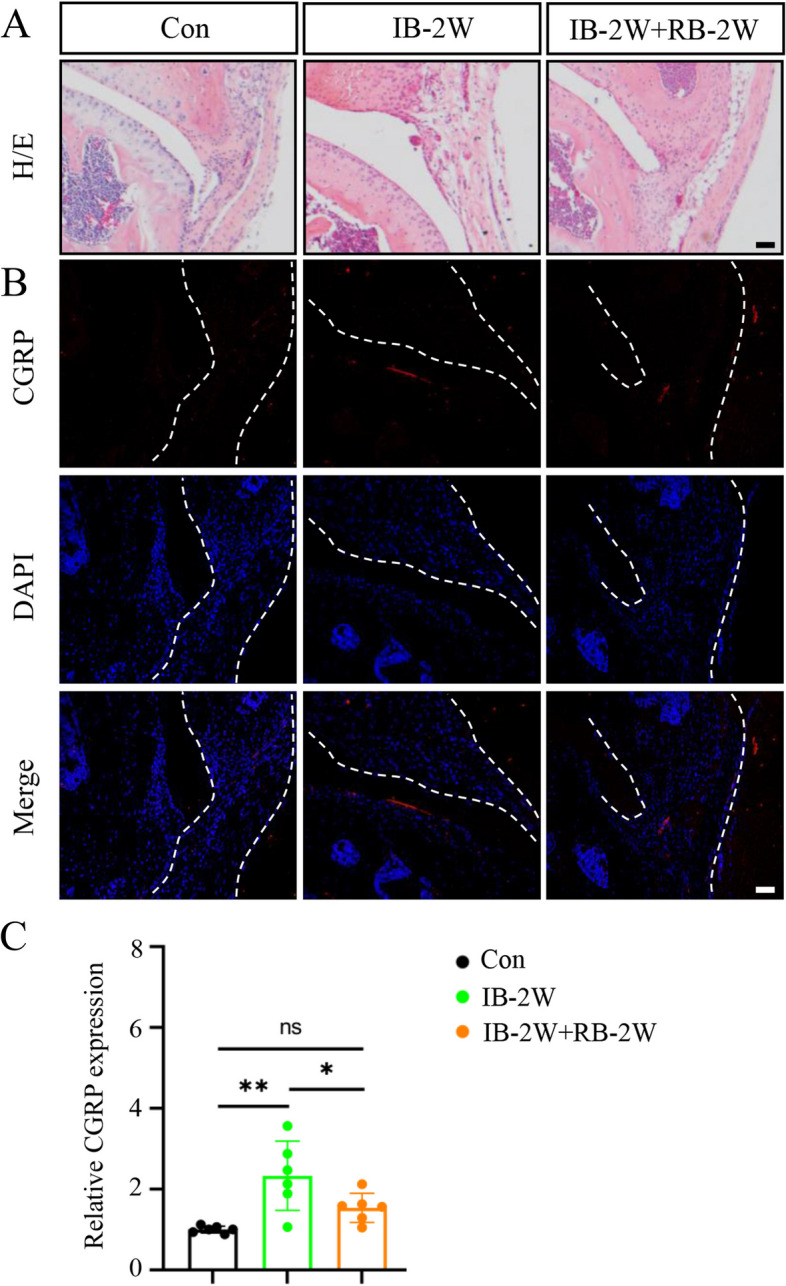
Joint remobilization ameliorates synovial sensory innervation in mice after short-term immobilization in mice. **A** Representative images of H/E staining images in knee joints from control, immobilization for 2 weeks, remobilization after 2-week immobilization. Scale bar, 50 mm. **B** Representative images of IF staining of CGRP staining (first row), DAPI staining (second row) and merge image (third row) in synovial tissue of knee joints of the indicated groups. The white dashed lines indicate the synovial tissue. Scale bar, 50 mm. **C** Quantitative data of expression of CGRP based on staining results in **B**. *N* = 6 biologically independent replicates per group. Results were expressed as mean ± standard deviation (sd). **P < 0.01. IB, immobilization; RB, remobilization

We next determined the effects of joint remobilization on recovery of OA lesions caused by joint immobilization. The results showed that the joint remobilization, which allows recovery of free activity and weight load of the knee joint, significantly ameliorated the synovial infiltration and pain induced by 2-week immobilization (Fig. 5A–H). Furthermore, the immobilization-induced increase in CGRP expression was significantly reduced by the joint remobilization (Fig. 6A–C).

## Discussion

In this study, we first demonstrate that the long-term joint immobilization causes significant joint pain and osteophyte formation in patients with extraarticular fractures. We then establish a mouse model of joint immobilization and determined the immobilization-induced effects on the knee joints at different times after immobilization. We demonstrate that the joint immobilization time dependently causes progressive OA-like lesions, including synovial infiltration and sensory innervation, vascular formation in synovium, joint pain, massive osteophyte formation, ossification of meniscus, and articular cartilage loss. We demonstrate that joint remobilization ameliorates some of the OA lesions and joint function in mice.

We find that the incidence of residual joint pain in patients increases with the prolongation of immobilization time in clinical practice. However, the radiographic changes in joint structure were not always consistent with the severity of pain [21]. In this study, we try to study this pathological change in the mouse joint immobilization model. Our results from mice reveal that synovial infiltration and thickening first appear after 2 weeks of joint immobilization, which later invades and grows into the joint cavity and eventually erodes the articular cartilage. With the extension of immobilization time, synovial fibrosis is gradually developed. At the same time, mice develop hyperalgesia in the knee joint, which confirms the clinical manifestation of increased VAS score or refractory joint pain after long-term joint immobilization. Peripheral neuronal sensitivity is an important factor in joint pain and synovitis, and synovial fibrosis can increase the sensitivity of local pain pathways [22–24]. The increase of nerve fibers containing the CGRP is closely related to peripheral neuron sensitivity [9]. In this study, we find that the expression of CGRP in synovial tissue is significantly increased by joint immobilization, which is correlated to the severity of joint pain in mice.

Cartilage degeneration is the most important pathological feature of OA [25]. We find that prolonged immobilization aggravates the articular cartilage erosion and proteoglycans loss. Previous studies have focused on the important role of mechanical stress in stimulating chondrocytes to secrete protease, leading to cartilage matrix degradation [26–28]. In this study, we find that the joint immobilization initially impacts the synovium, which first invades the joint cavity and then erodes the articular cartilage surface, followed by the loss of articular cartilage. This result supports the notion that synovial activation is an important factor in inducing cartilage degeneration and OA [29, 30]. Before cartilage degeneration occurs, the synovium has inflammatory cell infiltration, thickening of the synovial lining layer, and other activation manifestations [31]. A recent study reported that that IL-6, a key molecule that induces synovial activation, was produced in muscle tissue after joint immobilization, which may also be related to synovial activation after joint immobilization [32].

In this study, we find that significant osteophyte formation is observed on both sides of the femoral condyle and the edge of the tibial plateau even after 4 weeks of joint immobilization. With the extension of immobilization time, more osteophytes are formed. Some studies suggest that OA osteophytes are formed mainly through the intramembranous ossification process caused by stimulation of the periosteum and synovium [33–35]. Other studies suggest that progenitor cells at the junction of periosteum and synovium near articular cartilage are activated, thus forming osteophytes in cartilage and bone [10]. Synovial fibrosis is one of the characteristics of synovitis, which contributes to joint pain and stiffness [8]. Synovial fibrosis is characterized by excessive proliferation of FLS, differentiation to myofibroblast-like cells, and ECM synthesis [36]. Studies have shown that collagen type 1 is one of the key markers of both fibrocartilage formation and synovial fibrosis in OA [37]. In this study, we find that long-term immobilization causes severe synovial fibrosis and increases collagen expression level at the junction of synovial membrane and osteophyte, which may be related to osteophyte formation.

Our studies show that a short period of immobilization (i.e., 2 weeks) induces mild OA lesions, including synovial hyperplasia and pain sensitivity without causing obvious cartilage damage. These OA lesions are reversible and can be restored by remobilization of the knee joint. However, long period of immobilization causes severe structural damage of the articular cartilage, which is usually irreversible, thus resulting in a permanent damage to the joint. Previous studies have shown that long-term knee immobilization in young dogs led to changes in the biomechanical properties of cartilage, which was prone to degenerative changes in the joint [38]. Histological studies reported that immobilization led to articular chondrocyte apoptosis and matrix degradation, which was completely recovered when the joint moved again [5]. It is suggested that the time of tissue repair and joint immobilization should be balanced after clinical periarticular tissue injury, and joint functional exercise should be started as soon as possible, to restore joint function as much as possible and avoid irreversible degenerative OA.

## Conclusions

Joint immobilization caused multiple OA-like lesions in both mice and humans. Joint immobilization induced progressive sensory innervation, synovitis, osteophyte formation, and cartilage loss in mice, which can be partially ameliorated by remobilization. Information from this study may help clinicians develop novel strategies to prevent and reduce the complications of joint immobilization.

### Supplementary Information

**Additional file 1:** **Supplementary Figure 1.** Construction of the lower limb extension immobilization mouse model. (A) Figure A shows, from left to right, the ventral, back and lateral views.

**Additional file 2:** **Supplementary Table 1.** Antibody information.

## Data Availability

The datasets analyzed during the current study are available from the corresponding author on reasonable request.

## Abbreviations

OA: Osteoarthritis
VAS: Visual analogue scale
SPF: Specific pathogen-free
H/E: Hematoxylin-eosin
SO/FG: Safranin O/fast green
OARSI: Osteoarthritis Research Society International
IF: Immunofluorescence
HRP: Horseradish peroxidase
ANOVA: Analysis of variance
μCT: Micro-computed tomography
CGRP: Calcitonin gene-related peptide
