# Supplementary figures and images for: Pathological progress and remission strategies of osteoarthritic lesions caused by long-term joint immobilization
Source: Arthritis Res Ther. 2023 Dec 7;25:237. doi: 10.1186/s13075-023-03223-3 (PMC10702075; doi:10.1186/s13075-023-03223-3)

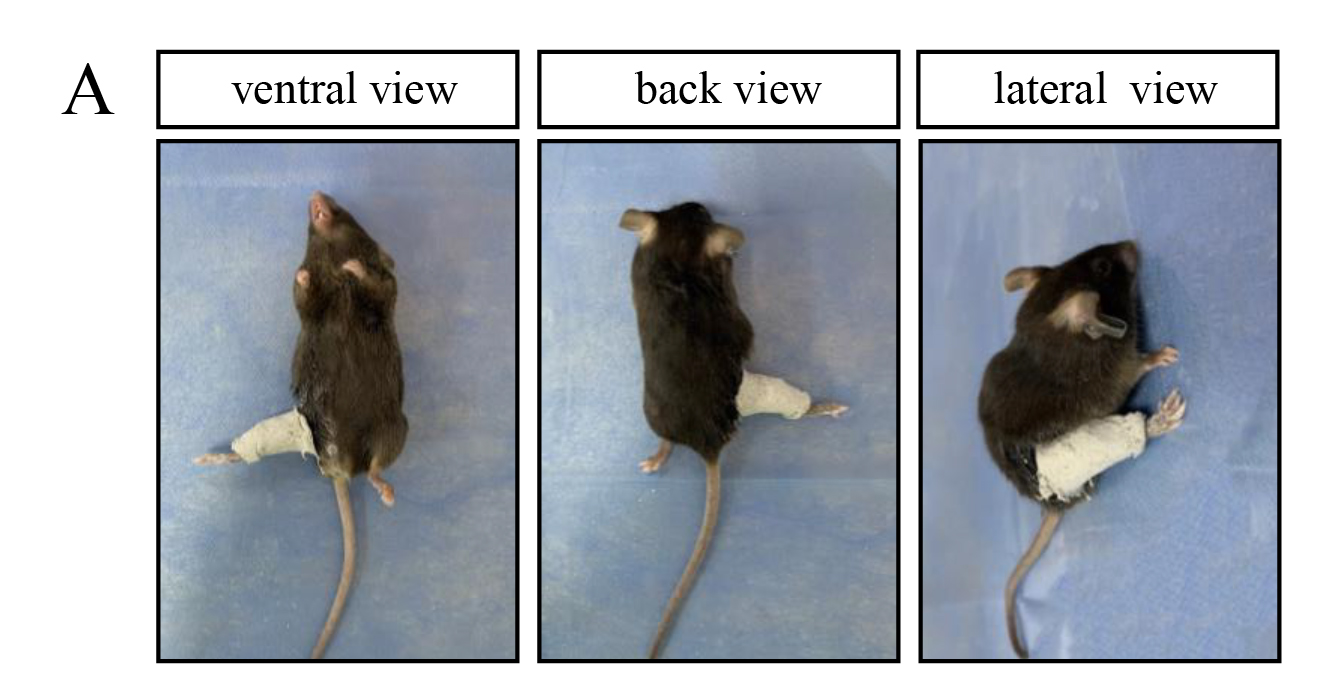

Supplement: Supplementary file 1 — Additional file 1: Supplementary Figure 1. Construction of the lower limb extension immobilization mouse model. (A) Figure A shows, from left to right, the ventral, back and lateral views. [file 13075_2023_3223_MOESM1_ESM.jpg]
