# Supplementary material for: Pathological progress and remission strategies of osteoarthritic lesions caused by long-term joint immobilization
Source: Arthritis Res Ther. 2023 Dec 7;25:237. doi: 10.1186/s13075-023-03223-3 (PMC10702075; doi:10.1186/s13075-023-03223-3)
Supplement: Supplementary file 2 — Additional file 2: Supplementary Table 1. Antibody information. [file 13075_2023_3223_MOESM2_ESM.docx]

**Supplementary Table 1:** Antibody information

| **Antibody** | **Company** | **catalog** | **Dilution** |
| --- | --- | --- | --- |
| CD31  CGRP | Abcam  Sigma | ab28364  c8198 | IHC (1:200)  IF (1:200) |
